# Special Issue “The Tight Junction and Its Proteins: From Structure to Pathologies”

**DOI:** 10.3390/ijms252313154

**Published:** 2024-12-07

**Authors:** Susanne M. Krug, Michael Fromm

**Affiliations:** Clinical Physiology/Nutritional Medicine, Charité—Universitätsmedizin Berlin, 12203 Berlin, Germany

Most tight junction (TJ) proteins build epithelial and endothelial barriers. Some, however, form paracellular ion or water channels. Besides barrier and channel properties, numerous other functions have gained increasing interest: TJ proteins can serve as receptors for pathogens and mediate immunological reactions. They are involved in several inflammatory diseases and bacterial infections. In cancer, they can mediate epithelial–mesenchymal transition, facilitating tumorigenesis and metastasis. They also serve as targets in tumor diagnostics and treatment. The Special Issue (SI) that we introduce here is a continuation of our two-volume Special Issue from 2020, “The Tight Junction and Its Proteins: More Than Just a Barrier” [1]. While the main title of our current Special Issue is unchanged, the second part differs. It reads “.... From Structure to Pathologies” in order to give credit to recent research progress on the molecular structure of TJ proteins, as well as on TJ-related pathologies.

This Special Issue contains 14 articles and provides a wide but far from complete spectrum of TJ research, which is given in comprehensive reviews elsewhere, of which we want to highlight two current ones. The first, “A short guide to the tight junction” [2], is exceptional in that seven well-known TJ researchers from different labs and countries have agreed on a unified opinion on TJ structure, function, and molecular components.

The other, “Biophysics of claudin proteins in tight junction architecture: Three decades of progress” [3], is particularly important as it presents a timeline of key milestones in the discovery, functional roles, and molecular structure of the claudin TJ proteins.

A unifying theme across the 14 articles of this Special Issue is the dynamic balance that TJs maintain between structural integrity and functional specialization.

In the brain, TJ proteins are crucial for blood–brain barrier integrity and are implicated in the pathogenesis of central nervous system disorders, which is emphasized along with their therapeutic potential in drug delivery and disease treatment in the review of Dithmer and colleagues [4]. Claudin-5, one of the main claudins of the blood–brain-barrier, may also serve as diagnostic biomarker for age-related changes in the blood–brain barrier and for Alzheimer disease, as plasma claudin-5 levels are elevated in Alzheimer’s disease and decrease with age [5]. The diverse spatial distributions and functional roles of TJ proteins during development were studied by Mak and Hammes during mouse forebrain development, where non-canonical localizations, such as claudin-3 in nuclear lamina, suggest that proteins contribute to epithelial dynamics and morphogenesis [6].

In the kidney, claudin-10 substantially influences gene expression in the thick ascending limb (TAL) of the loop of Henle, with differences observed between cortical (CTAL) and medullary (MTAL) regions. Claudin-10 is also particularly associated with genes involved in divalent cation reabsorption, which is important for renal function [7]. TJs also play critical roles in urinary acidification and sodium regulation. The study of Al-Shebel et al. reports that attempts to induce nephrocalcinosis by altering urinary pH in claudin-16-deficient mice did not lead to kidney calcifications. This indicates that urinary acidification alone does not prevent nephrocalcinosis in familial hypomagnesemia with hypercalciuria [8]. Modifications of body fluids do not only occur in the kidney but in all other organs, e.g., in sweat glands. In cholinergic urticaria, altered TJ protein distribution in these affects sweat composition, however, without compromising the barrier function [9].

In the gut, TJs also adapt while preserving essential barrier functions. The review of DiGuilio et al. states that micronutrients at supplemental levels strengthen TJs and enhance epithelial barrier function, underscoring their biological significance and offering potential therapeutic benefits in conditions like inflammation and COVID-19 [10]. Among such supplements, quercetin, a common plant flavonoid, enhances barrier integrity in porcine villus epithelium by modifying claudin protein expression but has no effect on Peyer’s patches [11]. This underlines the distinct functional properties of gut-associated lymphatic tissue and the surrounding epithelium. That, in immune cell-associated tissues and immune cells, TJ proteins may have distinct functionalities away from the commonly known ones is also suggested by Voges and colleagues. They report the dynamic expression of TJ proteins in immune cells, showing increased expression upon activation, and suggest that TJs may play a regulatory role in immune responses, with specific proteins being involved like LSR/angulin-1 and claudins interacting differently depending on the immune activation state [12].

Other functions of TJ proteins were observed on a cell level, where membrane tension influences the TJ and vice versa, as the absence of zonula occluden 2 protein (ZO-2) affects cellular mechanosensation by altering membrane tension and junctional protein recruitment. ZO-2 deficiency disrupts responses to substrate stiffness and topography, potentially exacerbating cellular transformations [13].

Targeting the molecular structure of TJs relies on understanding its molecular level. Computational studies and biophysical analyses are shedding light on the assembly and behavior of TJ proteins like claudin-10. Molecular simulations revealed that claudin-10a and -10b share similar channel architectures but differ in pore linings, resulting in their opposing charge selectivities [14].

Other computational studies, as reviewed by McGuiness and colleagues, detail the assembly and dynamics of claudin strands within TJs, deepening understanding of their ion channel functions and structural flexibility [15].

Several articles in this Special Issue highlight the translational potential of this research and suggest future directions, one of which involves novel drug delivery strategies. These are reviewed by Hansen at al. focusing on nanocarriers that might be applied via transdermal, ocular, pulmonary, and oral therapeutic delivery [16]. Methodological developments for studying barrier and transport functions are also progressing, as shown by Alija et al., who present a novel device that allows the continuous measurement of transepithelial flux in epithelial studies, thus revealing real-time changes in permeability. With this method, they validate TJ protein effects and provide insights into paracellular transport mechanisms [17].

Together, the findings of the articles within this Special Issue illuminate the multifaceted nature of TJs and their critical importance across disciplines. By combining fundamental science with therapeutic applications, TJ research continues to pave the way for innovations that address pressing health challenges and deepen our understanding of barrier function in complex tissues.
